# Intranasal insulin prevents anesthesia-induced hyperphosphorylation of tau in 3xTg-AD mice

**DOI:** 10.3389/fnagi.2014.00100

**Published:** 2014-05-30

**Authors:** Yanxing Chen, Xiaoqin Run, Zhihou Liang, Yang Zhao, Chun-ling Dai, Khalid Iqbal, Fei Liu, Cheng-Xin Gong

**Affiliations:** ^1^Department of Neurochemistry, New York State Institute for Basic Research in Developmental DisabilitiesStaten Island, NY, USA; ^2^Department of Neurology, Union Hospital, Tongji Medical College, Huazhong University of Science and TechnologyWuhan, Hubei, China; ^3^Department of Vascular Surgery, Union Hospital, Tongji Medical College, Huazhong University of Science and TechnologyWuhan, Hubei, China; ^4^Department of Neurology, The First Hospital of Jilin UniversityChangchun, China

**Keywords:** intranasal insulin, anesthesia, hyperphosphorylation of tau, brain insulin signaling, protein phosphatase 2A, tau protein kinases, Alzheimer’s disease, propofol

## Abstract

**Background:** It is well documented that elderly individuals are at increased risk of cognitive decline after anesthesia. General anesthesia is believed to be a risk factor for Alzheimer’s disease (AD). Recent studies suggest that anesthesia may increase the risk for cognitive decline and AD through promoting abnormal hyperphosphorylation of tau, which is crucial to neurodegeneration seen in AD.

**Methods:** We treated 3xTg-AD mice, a commonly used transgenic mouse model of AD, with daily intranasal administration of insulin (1.75 U/day) for one week. The insulin- and control-treated mice were then anesthetized with single intraperitoneal injection of propofol (250 mg/kg body weight). Tau phosphorylation and tau protein kinases and phosphatases in the brains of mice 30 min and 2 h after propofol injection were then investigated by using Western blots and immunohistochemistry.

**Results:** Propofol strongly promoted hyperphosphorylation of tau at several AD-related phosphorylation sites. Intranasal administration of insulin attenuated propofol-induced hyperphosphorylation of tau, promoted brain insulin signaling, and led to up-regulation of protein phosphatase 2A, a major tau phosphatase in the brain. Intranasal insulin also resulted in down-regulation of several tau protein kinases, including cyclin-dependent protein kinase 5, calcium/calmodulin-dependent protein kinase II, and c-Jun N-terminal kinase.

**Conclusion:** Our results demonstrate that pretreatment with intranasal insulin prevents AD-like tau hyperphosphorylation. These findings provide the first evidence supporting that intranasal insulin administration might be used for the prevention of anesthesia-induced cognitive decline and increased risk for AD and dementia.

## INTRODUCTION

Alzheimer’s disease (AD) is the most common form of dementia in adults, which accounts for an estimated 60 to 80% of dementia cases (Thies and Bleiler, 2013). The major brain pathological hallmarks of AD are extracellular senile plaques comprised predominantly of amyloid-β (Aβ) peptides, and intraneuronal neurofibrillary tangles (NTFs) comprised of abnormally hyperphosphorylated tau. The hyperphosphorylated tau loses its activity to bind to microtubules and to promote microtubule assembly, and instead disrupts microtubules (Iqbal et al., 1986, 2010).

The majority of AD cases have the sporadic form of the disease. Sporadic AD is multifactorial and may involve several different etiopathogenic mechanisms. It is well established that elderly individuals are at increased risk of cognitive decline after anesthesia or surgery (Moller et al., 1998; Rasmussen et al., 2003; Monk et al., 2008). Though the long-term effect of anesthesia on cognition is still under debate, anesthesia may accelerate preexisting but asymptomatic neurodegenerative changes in the brain and thus promote the development of AD (Papon et al., 2011). Evidence from animal models suggests that anesthetic exposure can increase Aβ plaque formation and tau hyperphosphorylation (Planel et al., 2007; Bianchi et al., 2008; Perucho et al., 2010; Run et al., 2010; Dong et al., 2012), and cause significant learning and memory deficits in aged rodents (Culley et al., 2004a, b; Bianchi et al., 2008; Le Freche et al., 2012; Shen et al., 2013).

Impaired brain insulin signaling pathway has been implicated in the development of AD (Correia et al., 2011; De Felice, 2013). We also found decreases in the levels and activities of several components of the insulin signaling pathway in AD (Liu et al., 2011). In agreement with the proposed role of insulin signaling in cognition, intranasal administration of insulin has been reported to improve memory in healthy humans (Benedict et al., 2004, 2007) and in individuals with mild cognitive impairment and AD (Reger et al., 2008a, b; Craft et al., 2012). Animal studies also show improved general behavioral performance and cognition in normal and diabetic mice after treatment with intranasal insulin (Francis et al., 2008; Marks et al., 2009). It has also been reported that insulin can affect with the stability, production, degradation and aggregation of Aβ, leading to reduced neurotoxicity (Qiu and Folstein, 2006; De Felice et al., 2009; de la Monte, 2012). However, whether intranasal insulin treatment prevents or ameliorates anesthesia-induced tau hyperphosphorylation, which is crucial to neurodegeneration, has not been reported.

In the present study, we treated 3xTg-AD mice, a commonly used transgenic model of AD which harbors three mutated transgenes (human PS1_M146V_, APP_SWE_, and tau_P301L_), with propofol, a commonly used intravenous anesthetic in clinical practice, and investigated the effects of intranasal insulin on propofol-induced hyperphosphorylation of tau. We found that insulin attenuated propofol-induced hyperphosphorylation of tau, which may be mainly through up-regulation of protein phosphatase 2A (PP2A) and down-regulation of several tau protein kinases.

## MATERIALS AND METHODS

### ANTIBODIES AND REAGENTS

Primary antibodies used in this study are listed in **Table 1**. Peroxidase-conjugated anti-mouse and anti-rabbit IgG were obtained from Jackson Immuno Research Laboratories (West Grove, PA, USA). The enhanced chemiluminescence (ECL) kit was from Pierce (Rockford, IL, USA). The ABC staining system was from Santa Cruz Biotechnology (Santa Cruz, CA, USA). Propofol was purchased from MP Biomedicals (Solon, OH, USA). Insulin (Humulin R U-100) was from Eli Lily (Indianapolis, IN, USA). Other chemicals were from Sigma-Aldrich (St. Louis, MO, USA).

**Table 1:** Primary antibodies used in this study.

Antibody	Type	Specificity	Phosphorylation sites	Source/reference
IRβ	Poly-	IRβ		Cell Signaling Technology, Danvers, MA
IGF-1Rβ	Poly-	IGF-1Rβ		Cell Signaling Technology
P-IRβ/IGF-1Rβ	Mono-	P-IRβ/IGF-1Rβ	Tyr1150/1151(IRβ), Tyr1135/1136 (IGF-1Rβ)	Cell Signaling Technology
IRS1	Poly-	IRS1		Cell Signaling Technology
IRS1 pS307	Poly-	P-IRS1	Ser307	Cell Signaling Technology
PI3K p85	Poly-	PI3K (p85)		Cell Signaling Technology
P-PI3K p85	Poly-	P-PI3K (p85)	Tyr458/Tyr199	Cell Signaling Technology
PDK1	Poly-	PDK1		Cell Signaling Technology
PDK1 pS241	Poly-	P-PDK1	Ser241	Cell Signaling Technology
AKT	Poly-	AKT		Cell Signaling Technology
AKT pS473	Poly-	P-AKT	Ser473	Cell Signaling Technology
AKT pT308	Poly-	P-AKT	Thr308	Cell Signaling Technology
GSK-3β pS9	Poly-	P-GSK-β	Ser9	Cell Signaling Technology
GSK-3α/β	Poly-	GSK-3β		Cell Signaling Technology
R134d	Poly-	Tau		Tatebayashi et al. (1999)
pS199	Poly-	P-tau	Ser199	Invitrogen, Grand Island, NY
pT205	Poly-	P-tau	Thr205	Invitrogen
pT212	Poly-	P-tau	Thr212	Invitrogen
pS214	Poly-	P-tau	Ser214	Invitrogen
pT231	Poly-	P-tau	Thr231	Invitrogen
pS262	Poly-	P-tau	Ser262	Invitrogen
12E8	Mono-	P-tau	Ser262/356	Dr. D. Schenk
PHF-1	Mono-	P-tau	Ser396/404	Dr. P. Davies
pS409	Poly-	P-tau	Ser409	Invitrogen
pS422 (R145)	Poly-	P-tau	Ser422	Pei et al. (1998)
CaMKII	Mono-	CaMKII		Promega, Madison, WI
CaMKII(pT286)	Poly-	P-CaMKII	Thr286	Promega
JNK	Poly-	JNK		Cell Signaling Technology
P-JNK	Poly-	P-JNK	Thr183/Tyr185	Cell Signaling Technology
ERK1/2	Mono-	ERK1/2		Cell Signaling Technology
P-ERK1/2	Poly-	P-ERK1/2		Cell Signaling Technology
CDK5	Poly-	CDK5		Santa Cruz Biotechnology
P35	Poly-	P35		Santa Cruz Biotechnology
PP2A-C	Mono-	PP2A-C		BD Bioscience, Palo Alto, CA
Methyl-PP2A-C	Mono-	Methyl-PP2A-C		Millipore, Temecula, CA
PP2A pY307	Poly-	P-PP2A	Tyr307	Santa Cruz Biotechnology, Santa Cruz, CA, USA
Anti-GAPDH	Poly-	GAPDH		Santa Cruz Biotechnology

### ANIMALS AND ANIMAL TREATMENTS

The breeding pairs of the homozygous 3xTg-AD mouse harboring PS1_M146V_, APP_Swe_, and tau_P301L_ transgenes and the wild type (WT) control mouse (a hybrid of 129/Sv and C57BL/6 mice) were initially obtained from Dr. F. M. LaFerla through Jackson Laboratory (New Harbor, 124 ME, USA), and the mice were bred in our institutional animal colony. Mice were housed (4~5 animals per cage) with a 12/12 h light/dark cycle and with ad libitum access to food and water. The housing, breeding, and animal experiments were in accordance with the approved protocol from our Institutional Animal Care and Use Committee, according to the PHS Policy on Human Care and Use of Laboratory animals (revised March 15, 2010).

**FIGURE 1:**
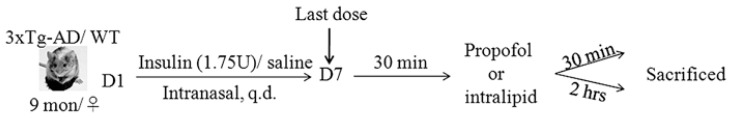
**Animal study design**.

The 3xTg-AD mice and WT mice (female, 9 months old) used for the present study were habituated to handling for 14 days prior to the experiment. Female mice were used because the female 3xTg-AD mice develop behavioral deficits faster than the male mice (Clinton et al., 2007). The selection of the age of 9 months was because the 3xTg-AD mice at this age show neurogenic and neuroplastic deficits but no NFTs or amyloid plaques and are cognitively impaired (Billings et al., 2005; Clinton et al., 2007; Mastrangelo and Bowers, 2008; Blanchard et al., 2010). Intranasal delivery was carried out manually without anesthesia while the mouse head was restrained in a supine position with the neck in extension, as described (Marks et al., 2009). A total of 1.75U/17.5 μl insulin or 0.9% saline (Veh 1) was delivered over both nares alternatively using a 10 μl Eppendorf pipetter. The mouse was held for an additional 5–10 s to ensure the fluid was inhaled. The successful nasal delivery by using this approach was confirmed by examination of ink in the autopsied brains after nasal delivery with ink using the same approach (data not shown). All mice were treated with insulin or, as a control, saline daily for seven consecutive days. Thirty minutes following the last dose, the mice were injected intraperitoneally (i.p.) with propofol dissolved in intralipid (250 mg/kg body weight) or the equivalent amount of intralipid (Veh 2), followed by sacrifice of the animals 30 min or 2 h later (**Figure 1**). The brains were removed immediately, and the rostral halves (separated coronally at the bregma level) of the mouse brains were dissected, flash frozen in dry ice, and stored at -80°C for biochemical analyses at a later date.

### WESTERN BLOT ANALYSIS

Mouse brain tissue was homogenized in pre-chilled buffer containing 50 mM Tris-HCl (pH 7.4), 50 mM GlcNAc, 20 μM UDP, 2.0 mM EGTA, 2 mM Na_3_VO_4_, 50 mM NaF, 20 mM Glycero-phosphate, 0.5 mM AEBSF, 10 μg/ml aprotinin, 10 μg/ml leupeptin, and 4 μg/ml pepstatin A. Protein concentrations of the homogenates were determined by using modified Lowery method. The samples were resolved in 10 or 12.5% SDS-PAGE and electro-transferred onto Immobilon-P membrane (Millipore, Bedford, MA, USA). The blots were then probed with primary antibody and developed with the corresponding horseradish peroxidase-conjugated secondary antibody and ECL kit (Pierce, Rockford, IL, USA). Densitometrical quantification of protein bands in Western blots were analyzed by using the TINA software (Raytest IsotopenmeBgerate GmbH, Straubenhardt, Germany).

### IMMUNOHISTOCHEMICAL STAINING

Floating sagittal sections were incubated at room temperature in 0.3% H_2_O_2_ for 30 min and then in 0.3% Triton X-100 for 15 min, washed in PBS, and blocked in a solution containing 5% normal goat serum and 0.1% Triton X-100 for 30 min. Sections were then incubated at 4°C with primary antibody overnight, followed by incubation with biotinylated secondary antibody and avidin/biotinylated horseradish peroxidase (Santa Cruz Biotechnology). The sections were stained with peroxidase substrate and then mounted on microscope slides (Brain Research Laboratories, Newton, MA, USA), dehydrated, and covered with cover slips.

### STATISTICAL ANALYSIS

For biochemical analyses, data were analyzed by one-way ANOVA followed by Tukey’s post hoc tests or unpaired two-tailed *t* tests, using Graph pad. All data are presented as means ± SEM, and *p* < 0.05 was considered statistically significant.

## RESULTS

### PROPOFOL EXACERBATES HYPERPHOSPHORYLATION OF TAU AT MULTIPLE SITES IN 3XTG-AD MICE

**FIGURE 2:**
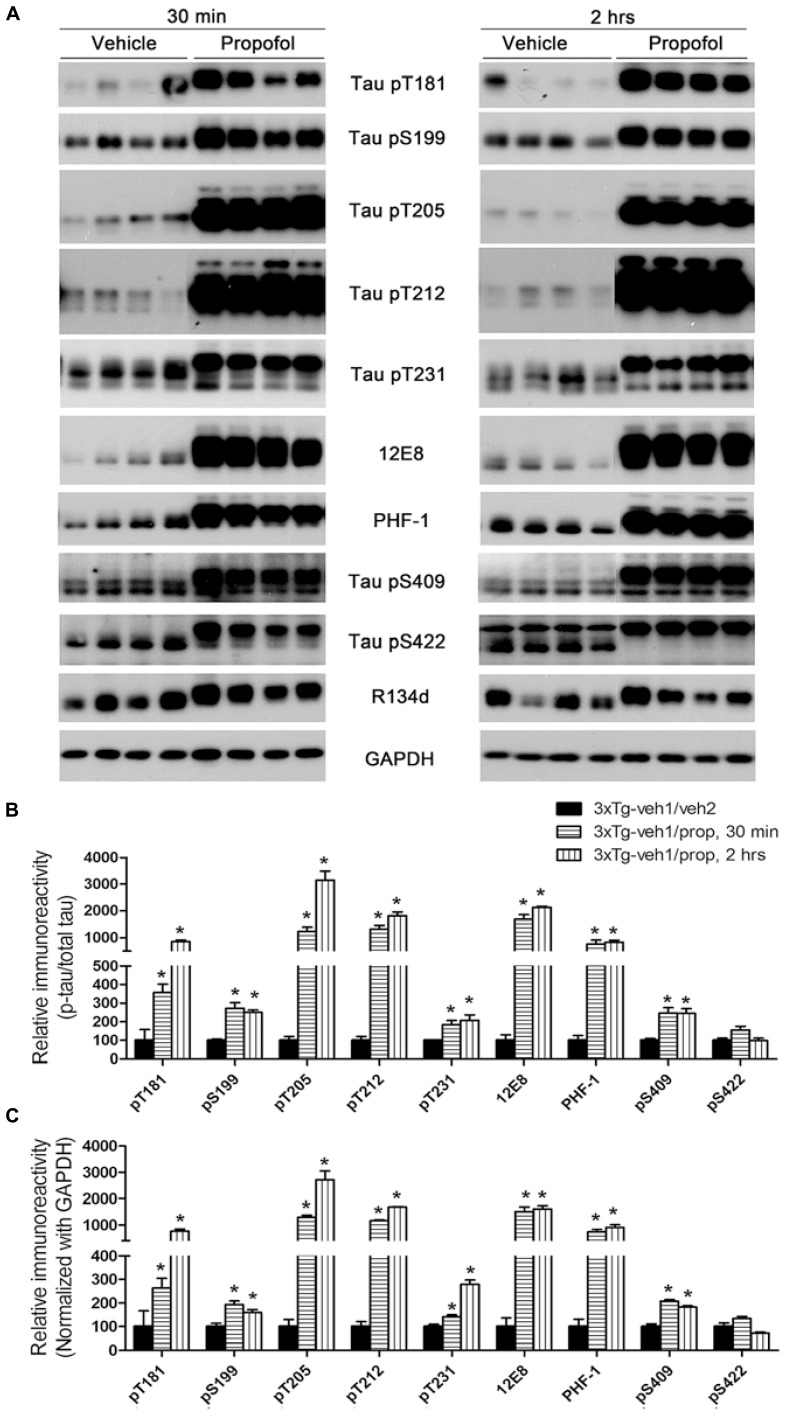
**Tau hyperphosphorylation at various AD-related sites in the brains of 3xTg-AD mice after anesthesia with propofol. (A)** Homogenates of the rostral halves of brains from 3xTg-AD mice sacrificed 30 min or 2 h following intraperitoneal injection of propofol were analyzed by Western blots developed with antibody R134d against total tau and several phosphorylation-dependent and site-specific tau antibodies, as indicated in the middle of the blots. **(B,C)** Densitometrical quantifications (mean ± SEM, *n* = 6/group) of the blots after being normalized with the corresponding total tau level **(B)** or with the GAPDH level **(C)**. The levels of the 3xTg-veh1/veh2 group were set to 100. *p < 0.05 vs. vehicle-injected 3xTg-AD mice.

We first verified the effect of propofol treatment on tau phosphorylation by using Western blots developed with phosphorylation-dependent and site-specific tau antibodies, which detect tau phosphorylation at Thr181, Ser199, Thr205, Thr212, Thr231, Ser262/356 (12E8 sites), Ser396/404 (PHF-1 sites), Ser409 and Ser422. As expected, we observed a marked increase in tau phosphorylation at all the above phosphorylation sites except Ser422 both 30 min and 2 h after propofol injection (**Figure 2**). Quantitative analyses indicated that the increase of tau phosphorylation was most dramatic at Thr181, Thr205, Thr212, Ser262/356 (12E8 sites), and Ser396/404 (PHF-1 sites; **Figures 2B, C**). The phosphorylation of tau at several sites was higher at 2 h than 30 min post anesthesia. Up-shift of the apparent gel mobility of tau, which is a well-established phenomenon of tau hyperphosphorylation (Kopke et al., 1993; Liang et al., 2008), was also seen (**Figure 2A**). These results confirmed marked increase in tau phosphorylation in mice anesthetized with propofol.

**FIGURE 3:**
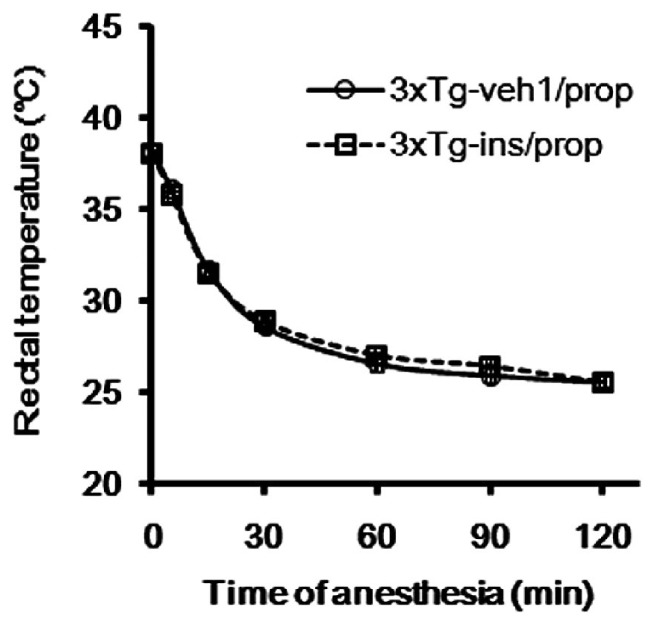
**Alteration of rectal temperature during anesthesia of the 3xTg-AD mice**.

Hypothermia is known to be a major factor underlying anesthesia-induced tau hyperphosphorylation (Planel et al., 2007; Run et al., 2009). Therefore, we determined the rectal temperature of the mice. We found that the average temperature dropped from around 38.2 to 28.6°C within 30 min after propofol injection (**Figure 3**), which is consistent with previous reports (Planel et al., 2007; Run et al., 2009). The average rectal temperature dropped further to 25.6°C 2 h after propofol injection when the mice had not woken from anesthesia. We did not prevent the anesthesia-induced hypothermia because the objective of this study is to evaluate intranasal insulin’s efficacy in the prevention of hyperphosphorylation of tau, rather than to investigate the role of hypothermia in propofol-induced brain changes, which have been reported previously (Planel et al., 2007; Run et al., 2009).

### INTRANASAL INSULIN ATTENUATES PROPOFOL-INDUCED TAU HYPERPHOSPHORYLATION

**FIGURE 4:**
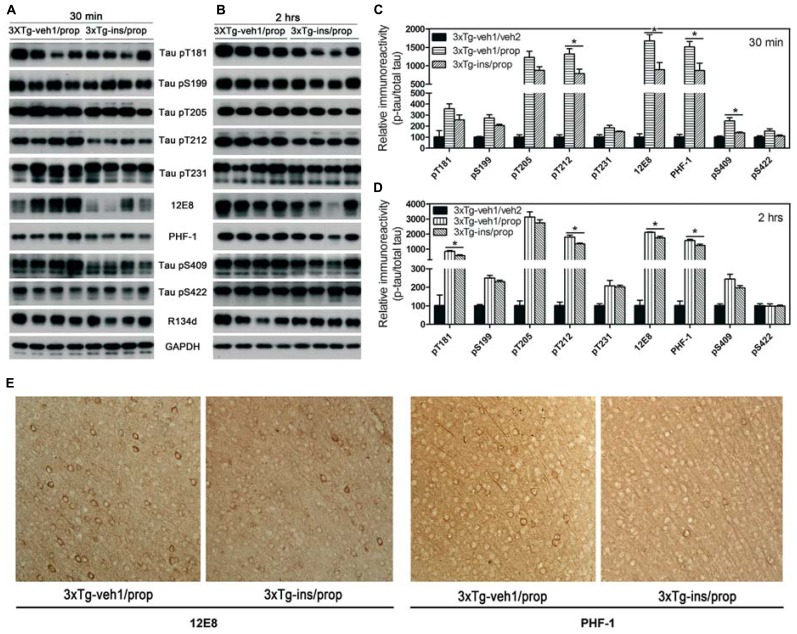
**Effect of intranasal insulin treatments on propofol-induced tau phosphorylation. (A,B)** Homogenates of the rostral halves of brains from 3xTg-veh1/prop and 3xTg-ins/prop mice sacrificed 30 min **(A)** or 2 h **(B)** following intraperitoneal injection of propofol were analyzed by Western blots developed with antibody R134d against total tau and several phosphorylation-dependent and site-specific tau antibodies, as indicated. **(C,D)** Densitometrical quantifications (mean ± SEM, *n* = 6/group) of the blots after being normalized with the corresponding total tau level. *p < 0.05. **(E)** Immunohistochemical staining of the brain tissue sections (frontal cortex) of mice sacrificed 30 min after propofol treatment. Monoclonal antibodies 12E8 and PHF-1 recognizes tau phosphorylated at Ser262/Ser356 and Ser396/Ser404, respectively.

Insulin is neuroprotective, and intranasal delivery is a non-invasive and effective way for insulin to reach the brain without affecting the peripheral blood glucose level (Reger et al., 2006). In order to investigate the effect of insulin on propofol-induced hyperphosphorylation of tau, we delivered insulin intranasally to 3xTg-AD mice for 7 days prior to the administration of propofol. We observed that mice receiving daily intranasal insulin (3xTg-ins/prop group) for 7 days before anesthesia had significantly lower phosphorylation level of tau at Thr212, Ser262/356 (12E8 sites), Ser396/404 (PHF-1 sites), and Ser409 at 30 min following propofol injection when compared to mice receiving saline (vehicle) only (3xTg-veh1/prop; **Figures 4A, C**). The phosphorylation level of tau at other epitopes studied (Thr181, Ser199, Thr205, Thr231, and ser422) was also lower in the insulin-treated group than the untreated group, but the decreases did not reach statistical significance (**Figure 4C**). Similar preventive role of insulin against propofol-induced tau phosphorylation was also seen 2 h following propofol injection (**Figures 4B, D**). The prevention of tau phosphorylation by insulin was confirmed immunohistochemically by using monoclonal antibodies 12E8 and PHF-1 against phosphorylated tau. We found that the number of strongly stained neurons in the mouse brains was markedly reduced in the 3xTg-ins/prop group as compared to the 3xTg-veh1/prop group (**Figure 4E**). These results indicate that intranasal insulin administration attenuates propofol-induced tau hyperphosphorylation in the mouse brain. The insulin’s action in attenuation of propofol-induced tau hyperphosphorylation was not related to hypothermia because no difference in the body temperature between the insulin-treated and untreated mice was observed (**Figure 3**).

### INTRANASAL INSULIN ENHANCES BRAIN INSULIN SIGNALING

**FIGURE 5:**
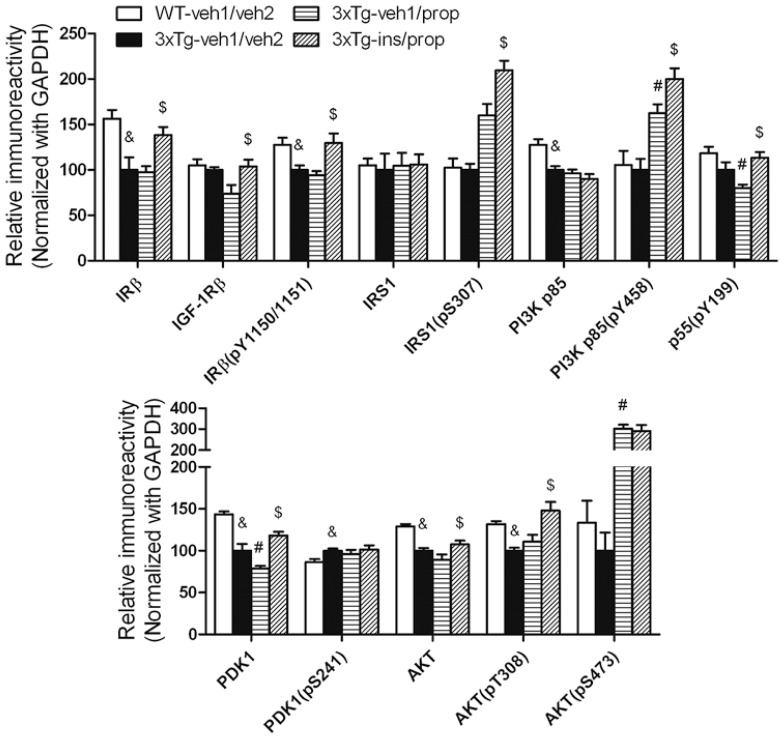
**Effect of propofol and intranasal insulin treatment on brain insulin signaling**. Homogenates of the rostral halves of brains from mice sacrificed 30 min following propofol injection were analyzed by Western blots developed with the indicated antibodies. The blots were then quantified densitometrically, and the data are presented as mean ± SEM (*n* = 6/group), where the values of the 3xTg-veh1/veh2 group were set as 100%. ^&^*p* < 0.05 vs. WT-veh1/veh2 group; ^#^*p* < 0.05 vs. 3xTg-veh1/veh2 group; ^$^*p* < 0.05 vs. 3xTg-veh1/prop group.

To understand the possible mechanism of the beneficial effect of intranasal insulin treatment on tau hyperphosphorylation, we investigated its effect on insulin signaling in the mouse brain by comparing the level and activation of each component of the signaling pathway, including insulin receptor β (IRβ), insulin-like growth factor-1 receptor β (IGF-1Rβ), insulin receptor substrate-1 (IRS-1), phosphatidylinositide 3-kinases (PI3K), 3-phosphoinositide-dependent protein kinase-1 (PDK1) and protein kinase B (AKT). The activation of these proteins was assessed by measuring their phosphorylation levels at the activity-dependent sites. We found that insulin signaling pathway was disturbed in the brains of 3xTg-AD mice (**Figures 5A, B**, 3xTg-veh1/veh2 vs. WT-veh1/veh2). Propofol further disturbed the insulin signaling, as evidenced by down-regulation of the level of PI3K p55 pY199 and PDK1 and up-regulation of the level of PI3K p85 pY458 and AKT pS473 (**Figures 5A, B**). The dramatic increase in AKT pS473 might result from a cross-talk with other signaling pathway activated by propofol. We observed that intranasal delivery of insulin for 7 days prior to the administration of propofol enhanced the insulin signaling transduction in the brain. The levels of IRβ, IGF-1Rβ, IRβ pY1150/1151, IRS1 pS307, PI3K p85 pY458, PI3K p55 pY199, PDK1, AKT, and AKT pT308 were all up-regulated in the 3xTg-ins/prop mice as compared to the 3xTg-veh1/prop mice (**Figure 5**). These results indicate that intranasal insulin promotes brain insulin signaling.

### INTRANASAL INSULIN UP-REGULATES PP2A IN THE BRAIN

**FIGURE 6:**
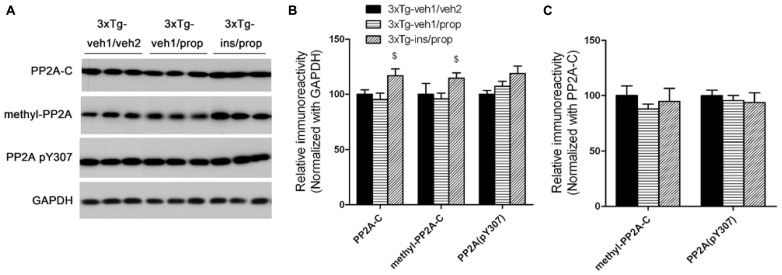
**Effect of propofol and intranasal insulin treatments on PP2A. (A)** Homogenates of the rostral halves of mouse brains were analyzed by Western blots developed with antibodies indicated at the left side of the blots. **(B,C)** Densitometrical quantifications (mean ± SEM, *n* = 6/group) of the blots after normalization with the GAPDH levels **(B)** or with the PP2A-C levels **(C)**. ^$^*p* < 0.05 vs. 3xTg-veh1/prop group.

Down-regulation of PP2A, a major tau phosphatase in the brain (Liu et al., 2005), was shown to underlie anesthesia-induced hyperphosphorylation of tau (Planel et al., 2007). To investigate whether PP2A also mediates insulin’s activity to attenuate propofol-induced tau hyperphosphorylation, we determined the level of the catalytic subunit of PP2A (PP2A-C) and its methylation, which enhances PP2A activity (Guenin et al., 2008), and tyrosine-phosphorylation at Tyr307, which inhibits its activity (Chen et al., 1992). We observed that intranasal insulin treatment led to a significant increase in the level and the methylation of PP2A in the mouse brain (**Figures 6A, B**). However, the net PP2A methylation, as quantified after normalization with the PP2A-C level, was not increased (**Figure 6C**), suggesting that the increased total PP2A methylation is due to the increase of PP2A-C level. These results suggest that intranasal insulin might attenuate propofol-induced tau hyperphosphorylation through an increase of methylated PP2A-C and thus its activity in the brain.

### ACTIVITIES OF SEVERAL TAU KINASES ARE ALTERED IN THE MOUSE BRAIN AFTER PROPOFOL AND INTRANASAL INSULIN TREATMENTS

**FIGURE 7:**
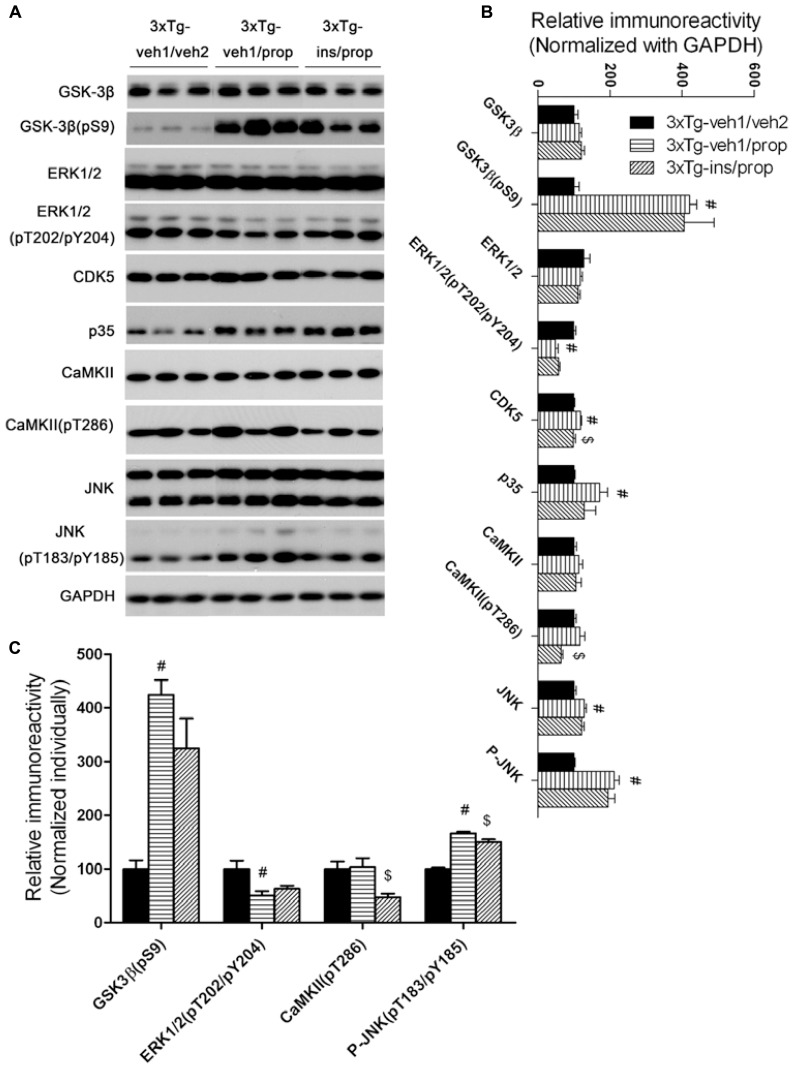
**Effect of propofol and intranasal insulin treatments on tau kinases. (A)** Homogenates of the rostral halves of mouse brains were analyzed by Western blots developed with antibodies indicated at the left side of the blots. **(B,C)** Densitometrical quantifications (mean ± SEM, *n* = 6/group) of the blots after being normalized with the GAPDH levels **(B)** or with the levels of the corresponding total protein kinase level **(C)**. ^#^*p* < 0.05 vs. 3xTg-veh1/veh2 group; ^$^*p* < 0.05 vs. 3xTg-veh1/prop group.

Besides PP2A, tau phosphorylation is also regulated by several protein kinases (Wang and Liu, 2008). The levels of several tau protein kinases have been reported to be altered after anesthesia (Planel et al., 2007; Run et al., 2009). We therefore determined the levels of the total and the activated form of several tau protein kinases, including glycogen synthase kinase-3β (GSK-3β), mitogen-activated protein kinase/extracellular signal-regulated kinase (MAPK/ERK), cyclin-dependent kinase 5 (cdk5), calcium/calmodulin-dependent protein kinase II (CaMKII), and c-Jun N-terminal kinase (JNK). Consistent with previous studies (Planel et al., 2007; Run et al., 2009), we found a dramatic increase in the inhibitory Ser9 phosphorylation of GSK-3β and moderate alterations of a few other tau kinases after anesthesia with propofol (**Figure 7**). Intranasal insulin treatment resulted in mild to moderate reductions of CDK5, CaMKII pT286, and JNK pT184/pY185 in the propofol-treated mouse brains. These results suggest that the insulin-induced reduction of these three tau kinases might also contribute to insulin’s role in attenuation of propofol-induced tau hyperphosphorylation.

## DISCUSSION

Tau is the major microtubule-associated protein of mature neurons. Its major known physiological activity is to promote the assembly of tubulin into microtubules and to stabilize microtubule structure. Abnormally hyperphosphorylated tau fails to bind to tubulin and also gains a toxic activity of disrupting microtubules (Iqbal et al., 1986; Alonso et al., 1994). Therefore, abnormal hyperphosphorylation of tau appears to be crucial to neurodegeneration in AD and other tauopathies (Gong et al., 2010). The vital role of tau in neurodegeneration have been further supported by several recent in vivo studies showing that tau knockout reduces or eliminates neurodegeneration and behavioral deficits in transgenic mouse models of AD (Roberson et al., 2007; Gomez de Barreda et al., 2010).

Many epidemiological studies have demonstrated that general anesthesia induces memory loss and increases the risk for dementia and AD (Papon et al., 2011). In an effort to understand the possible underlying mechanisms, several studies have found that both intravenous and inhalational anesthetics induce hyperphosphorylation of tau in the brain (Ikeda et al., 2007; Planel et al., 2007; Run et al., 2009, 2010; Le Freche et al., 2012). These studies suggest an important molecular mechanism by which anesthesia may induces memory loss and increases the risk for dementia and AD through promoting abnormal hyperphosphorylation of tau and consequently neurodegeneration (Planel et al., 2009; Run et al., 2009; Le Freche et al., 2012).

Planel et al. (2007) reported that anesthetics induced hyperphosphorylation of tau as a consequence of PP2A inhibition by hypothermia. Subsequently, two distinct underlying mechanisms were found: one associating with activation of stress-activated protein kinases and the other resulting from anesthesia-induced hypothermia (Run et al., 2009; Whittington et al., 2011). In consistent with previous reports, we found, in the present study, a marked increase in tau phosphorylation at several AD-related sites after 3xTg-AD mice were anesthetized with propofol. Furthermore, tau hyperphosphorylation was accompanied with activation of JNK but inhibition of GSK-3β and ERK after anesthesia with propofol. These findings are consistent with previous observations showing activation of JNK and marked inhibition of GSK-3β and ERK in WT mice after anesthesia (Planel et al., 2007, 2009; Run et al., 2009; Le Freche et al., 2012).

Drug administration into the brain through intranasal delivery, which was first introduced by Dr. W. H. Frey, bypasses the blood brain barrier and has been used successfully in animal studies and clinical trials in humans (Reger et al., 2006, 2008b; Francis et al., 2008; Hanson and Frey, 2008; Marks et al., 2009; Craft et al., 2012; Yang et al., 2013). Intranasal administration of insulin has an additional advantage that it does not interfere with the insulin level or glucose metabolism in the periphery (Reger et al., 2006). Thus, in the present study we selected this approach to investigate whether insulin prevents or ameliorates anesthesia-induced tau hyperphosphorylation. We found that daily administration of insulin for a week significantly prevented propofol-induced tau hyperphosphorylation at several AD-related sites. Furthermore, the insulin’s preventive role might result from its promotion of brain insulin signaling and PP2A and down-regulation of several tau protein kinases, CDK5, CaMKII, and JNK. Although hypothermia was observed in the 3xTg-AD mice after anesthesia with propofol, the insulin’s preventive role does not seem to be associated with hypothermia because pre-treatment of mice with intranasal insulin did not affect propofol-induced hypothermia. Our present findings provide important experimental evidence supporting that intranasal insulin treatment might be used for preventing anesthesia-induced risk for memory loss and dementia.

In summary, we have found that anesthesia with propofol exacerbated hyperphosphorylation of tau at multiple AD-related phosphorylation sites in the brain of 3xTg-AD mice. These findings provide experimental evidence supporting the role of anesthesia in increasing the risk for dementia and AD in vulnerable individuals and demonstrate that anesthesia could be a significant factor for AD in those elderly individuals who have received general anesthesia. Furthermore, we found, for the first time, that intranasal administration of insulin for a week prior to anesthesia significantly prevented propofol-induced tau hyperphosphorylation and enhanced brain insulin signaling. These findings provide the first evidence supporting that intranasal insulin might be a promising treatment for prevention of anesthesia-induced memory loss and increased risk for AD and dementia.

## AUTHOR CONTRIBUTIONS

Conception of the research: Cheng-Xin Gong, Xiaoqin Run, Yanxing Chen, Zhihou Liang, Khalid Iqbal, and Fei Liu. Performing experiments: Yanxing Chen, Yang Zhao, and Chun-ling Dai. Analyses and interpretation of results: Yanxing Chen, Xiaoqin Run, Zhihou Liang, Yang Zhao, Chun-ling Dai, Khalid Iqbal, Fei Liu, and Cheng-Xin Gong. Drafting of the manuscript: Yanxing Chen. Critical revision of the manuscript: Cheng-Xin Gong, Yanxing Chen, Xiaoqin Run, Khalid Iqbal, and Fei Liu.

## Conflict of Interest Statement

The authors declare that the research was conducted in the absence of any commercial or financial relationships that could be construed as a potential conflict of interest.
